# Microtiter plate cultivation of oleaginous fungi and monitoring of lipogenesis by high-throughput FTIR spectroscopy

**DOI:** 10.1186/s12934-017-0716-7

**Published:** 2017-06-09

**Authors:** Gergely Kosa, Achim Kohler, Valeria Tafintseva, Boris Zimmermann, Kristin Forfang, Nils Kristian Afseth, Dimitrios Tzimorotas, Kiira S. Vuoristo, Svein Jarle Horn, Jerome Mounier, Volha Shapaval

**Affiliations:** 1 Faculty of Science and Technology, Norwegian University of Life Sciences, Postbox 5003, 1432 Ås, Norway; 2 Nofima AS, Osloveien 1, 1430 Ås, Norway; 3 Faculty of Chemistry, Biotechnology and Food Science, Norwegian University of Life Sciences, Postbox 5003, 1432 Ås, Norway; 4 Université de Brest, EA3882 Laboratoire Universitaire de Biodiversité et Ecologie Microbienne, IBSAM, ESIAB, Technopôle Brest Iroise, 29280 Plouzané, France

**Keywords:** Microcultivation, Oleaginous fungi, Fatty acid analysis, GC-FID, High-throughput FTIR spectroscopy, PLS regression

## Abstract

**Background:**

Oleaginous fungi can accumulate lipids by utilizing a wide range of waste substrates. They are an important source for the industrial production of omega-6 polyunsaturated fatty acids (gamma-linolenic and arachidonic acid) and have been suggested as an alternative route for biodiesel production. Initial research steps for various applications include the screening of fungi in order to find efficient fungal producers with desired fatty acid composition. Traditional cultivation methods (shake flask) and lipid analysis (extraction-gas chromatography) are not applicable for large-scale screening due to their low throughput and time-consuming analysis. Here we present a microcultivation system combined with high-throughput Fourier transform infrared (FTIR) spectroscopy for efficient screening of oleaginous fungi.

**Results:**

The microcultivation system enables highly reproducible fungal fermentations throughout 12 days of cultivation. Reproducibility was validated by FTIR and HPLC data. Analysis of FTIR spectral ester carbonyl peaks of fungal biomass offered a reliable high-throughput at-line method to monitor lipid accumulation. Partial least square regression between gas chromatography fatty acid data and corresponding FTIR spectral data was used to set up calibration models for the prediction of saturated fatty acids, monounsaturated fatty acids, polyunsaturated fatty acids, unsaturation index, total lipid content and main individual fatty acids. High coefficients of determination (R^2^ = 0.86–0.96) and satisfactory residual predictive deviation of cross-validation (RPD_CV_ = 2.6–5.1) values demonstrated the goodness of these models.

**Conclusions:**

We have demonstrated in this study, that the presented microcultivation system combined with rapid, high-throughput FTIR spectroscopy is a suitable screening platform for oleaginous fungi. Sample preparation for FTIR measurements can be automated to further increase throughput of the system.

**Electronic supplementary material:**

The online version of this article (doi:10.1186/s12934-017-0716-7) contains supplementary material, which is available to authorized users.

## Background

Generally, a microorganism is considered oleaginous if the lipid content exceeds 20% of its dry weight, while up to 70% lipid content has been reported in the literature [1]. To achieve such high lipid content, microorganisms need to be cultivated in excess of a carbon source, while other nutrients such as nitrogen should be present in limiting concentration, i.e. in a high carbon-to-nitrogen ratio. Oleaginous microorganisms respond to nitrogen depletion by accumulating carbon in the form of triacylglycerol (TAG) in distinct lipid bodies. Oleaginous species can be found among yeasts, filamentous fungi and microalgae, while bacteria usually produce polyhydroxybutyrate and polyhydroxyalkanoate as storage polymers [1].

Microbial oils or single cell oils (SCO) are important sources of high-value polyunsaturated fatty acids (PUFA) for human consumption as nutraceuticals. Plants do not produce PUFA longer than C18 (trienoic acids), while fish oil has several disadvantages, such as odor, accumulated toxic compounds, and overfishing [1, 2]. The commercially produced microbial oils contain high percentage of polyunsaturated fatty acids (PUFA). For example, omega-6 PUFAs, such as gamma-linolenic acid (18:3, GLA) and arachidonic acid (20:4, ARA) have been produced by the filamentous fungi *Mucor circinelloides* and *Mortierella alpina,* respectively.

Initial steps in microbial lipid research involve the screening for efficient production strains, which are either genetically modified organisms or natural isolates, and media optimization for subsequent scale-up experiments. When a large number of strains or cultivation conditions have to be tested, a high throughput screening (HTS) system is required, which can yield reproducible and scalable results. In order to increase throughput compared to traditional shake flasks, miniaturization of cultivations by microtiter plates or microbioreactors is desired [3–5]. HTS of filamentous fungi in microtiter plates is a challenging task because (a) there is a substantial risk of cross-contamination between individual cultivations, (b) highly viscous fermentation broth can cause oxygen transfer limitation and local inhomogeneity, and (c) excessive wall growth may take place, which favors sporulation.

For the screening of oleaginous microorganisms in microplates, a rapid, accurate lipid analysis is desired. Extraction, transesterification and gas chromatography (GC) is time-consuming, expensive and requires sample preparation and analysis that creates toxic wastes [6–8]. Rapid, non-invasive methods for SCO analysis involve fluorescence based measurements, near-infrared spectroscopy (NIR), Fourier transformed infrared spectroscopy (FTIR) and Raman spectroscopy [9]. FTIR spectroscopy has been successfully applied in recent years for microbial lipid research in yeast [8, 10, 11], microalgae [7, 12–14] and filamentous fungi [15]. FTIR spectroscopy is extremely versatile since it enables the overall characterization of the biochemical composition of intact cells, including proteins, lipids, and carbohydrates [11]. For that reason, FTIR spectroscopy is widely used for the rapid differentiation and identification of microorganisms [16–19]. The main advantages of FTIR spectroscopy are that (a) several compounds can be measured simultaneously, (b) the method is rapid since little or no sample preparation is required for spectral acquisition, (c) it is chemical-free, (d) it can be used for HTS and for real-time bioprocess monitoring, (e) and even spatial information can be obtained by the use of FTIR microspectroscopy systems [20, 21]. Since infrared spectra are highly complex, with many overlapping signals, multivariate data analysis is required to gain useful information [17, 22].

The aim of this study was to introduce the Duetz microtiter plate system (Duetz-MTPS) combined with HTS-FTIR spectroscopy as a high-throughput analytical platform for screening and monitoring of oleaginous fungi. In order to demonstrate the suitability of the system, we have used three fungal species and incubated them for 12 days under different temperatures. We have monitored lipogenesis of the fungal fermentations in microplates by high-throughput FTIR spectroscopy and GC reference analysis.

## Methods

### Fungal strains

Three oleaginous filamentous fungi were used in this study: *Mucor* *circinelloides* VI 04473 (Norwegian School of Veterinary Science; Oslo, Norway), *Umbelopsis isabellina* UBOCC-A-101350 (Université de Bretagne Occidentale Culture Collection; Plouzané, France) and *Penicillium glabrum* FRR 4190 (Commonwealth Scientific and Industrial Research Organisation; North Ryde, Australia).

### Media and growth conditions

Cultivation of fungi was first performed on agar media to obtain spores for the inoculation and then in nitrogen-limited liquid medium in order to stimulate lipid accumulation. For spore inoculum preparation the following agar media were used: malt extract agar (MEA) for *M. circinelloides* and *P. glabrum* and potato dextrose agar (PDA) for *U. isabellina*. MEA was prepared by dissolving 30 g malt extract (Merck, Germany), 5 g peptone (Amresco, USA) and 15 g agar powder (VWR Chemicals, Belgium) in 1 L distilled water and autoclaved at 115 °C for 10 min. PDA was prepared by dissolving 39 g potato dextrose agar (VWR Chemicals, Belgium) in 1 L distilled water and autoclaved at 121 °C for 15 min. All agar cultivations were performed for 7 days at 25 °C. Spores were harvested with a bacteriological loop from agar plates after the addition of 10 mL sterile physiologic salt solution. Spore concentrations were measured with hemocytometer (Fuchs-Rosenthal, Hausser Scientific Company, USA) and a DM6000B microscope (Leica Microsystems, Germany) and spore suspensions were diluted to 3.6 × 10^6^ spore mL^−1^.

The broth medium was prepared according to the protocol described in Kavadia et al. [23] with modifications (g L^−1^): glucose 80, yeast extract (Oxoid, England) 3, KH_2_PO_4_ 7, Na_2_HPO_4_ 2, MgSO_4_·7H_2_O 1.5, CaCl_2_·2H_2_O 0.1, FeCl_3_·6H_2_O 0.008, ZnSO_4_·7H_2_O 0.001, CoSO_4_·7H_2_O 0.0001, CuSO_4_·5H_2_O 0.0001, MnSO_4_·5H_2_O 0.0001. Chemicals (except yeast extract) were purchased from Merck (Germany). Liquid medium was autoclaved for 15 min at 121 °C. The pH of the medium was 6.05 after sterilization. Cultivation in liquid medium was performed in the Duetz-MTPS (Enzyscreen, Netherlands), consisting of 24-square polypropylene deep well plates, low-evaporation sandwich covers and extra high cover clamps [24], which were mounted in two Innova 40R refrigerated desktop shakers (Eppendorf, Germany). Autoclaved and dried microtiter plates were filled with 2.5 mL of sterile liquid medium by using the Stepper 411 adjustable repeater pipette (Socorex, Switzerland). Each well was inoculated with 50 µL fungal spore suspension. Cultivations were performed for 12 days at 20 and 30 °C at 300 rpm agitation speed (circular orbit 0.75″ or 19 mm). Each day, one plate was removed from both shakers for analysis.

### Experimental design

All microplates were prepared in the following scheme: the first eight wells were inoculated with *M. circinelloides*, the second eight wells with *U. isabellina* and the last eight wells with *P. glabrum*. For each strain, fungal biomass of the first three wells, considered as biological replicates (210 samples in total), was used for lipid analysis by HTS-FTIR spectroscopy. Supernatant of the same wells, as well as the starting growth medium (215 samples in total), was used for glucose and protein analyses by HPLC and colorimetric assay. Finally, the merged biomass from the other five wells (70 samples in total) was used for lipid analysis by gas chromatography (GC).

### Bright-field and fluorescent microscopy

Morphology of the filamentous fungi was examined with a DM6000B microscope (Leica Microsystems, Germany). Microscopic pictures were obtained with an Evolution MP camera kit (Media Cybernetics, USA). A Nile-red staining solution was prepared by dissolving 1 mg Nile-red crystals (Sigma-Aldrich, Germany) in 1 mL ethanol. Then, 10 µL Nile-red solution was dried onto a glass side, the biomass was added and covered with a glass coverslip. Nile-red stained samples were incubated for 1 h at 4 °C in the dark and images were captured using a 490 nm excitation/530 nm emission wavelength filter cube (Leica Microsystems, Germany).

### Preparation of supernatant and biomass

The supernatant was separated from the fungal biomass by transferring 2 mL fermentation broth with plastic Pasteur pipettes into Eppendorf tubes and the subsequent centrifugation at 13,000 rpm for 20 min at 4 °C. Fungal biomass from Eppendorf tubes were washed three times with cold distilled water and filtered under vacuum using a Whatman No. I filter paper (GE Whatman, USA). All samples were stored at −20 °C until analysis.

### Preparation of fungal biomass for FTIR analysis

The washed fungal biomass (approx. 50 µL per sample) was homogenized in 96-square deepwell plates with 500 µL distilled water using a modular liquid handling robot [25] with integrated 2 mm single-pin Q55 sonicator (Qsonica, USA). The sonication was performed in a pulse regime with 15 s sonication time and 5 s washing time. Total sonication time for *U. isabellina*, *M. circinelloides*, and *P. glabrum* was 30 s, 1 min and 1.5 min, respectively. *P. glabrum* biomass cultivated at 20 °C was manually sonicated for 2 min, due to a rigid pellet structure, which was difficult to homogenize with the robotic system.

### FTIR spectroscopy

FTIR analysis of the sonicated fungal biomass was performed using the High Throughput Screening eXTension (HTS-XT) unit coupled to the Vertex 70 FTIR spectrometer (both Bruker Optik, Germany) in transmission mode. From each suspension, 8 µL were transferred to an IR-light-transparent silicon 384-well microplate (Bruker Optik, Germany) in three technical replicates. Samples were dried at room temperature for 2 h to form films that were suitable for FTIR analysis. The spectra were recorded in the region between 4000 and 500 cm^−1^ with a spectral resolution of 6 cm^−1^ and an aperture of 5.0 mm. For each spectrum, 64 scans were averaged. Each spectrum was recorded as the ratio of the sample spectrum to the spectrum of the empty microplate. In total, 210 samples were measured and 630 FTIR spectra were obtained.

### Glucose analysis

Glucose was quantified using an UltiMate 3000 UHPLC system (Thermo Scientific, USA) equipped with RFQ-Fast Acid H + 8% (100 × 7.8 mm) column (Phenomenex, USA) and coupled to a refractive index (RI) detector. Samples were diluted ten times before analysis, filter sterilized and were subsequently eluted isocratically at 0.6 mL min^−1^ flow rate in 12 min with 5 mM H_2_SO_4_ mobile phase at 85 °C column temperature.

### Protein analysis

Protein concentration in sample of supernatants was determined with a Bradford-method based colorimetric assay (Bio-Rad Protein Assay, USA) according to the microplate protocol. Absorbance was measured at 595 nm with a SPECTROstar Nano UV/Vis microplate reader (BMG Labtech, Germany). A calibration curve was prepared with media containing different amount of yeast extract.

### Preparation of fungal biomass for FAME extraction and GC analysis

The fermentation broth from the other five wells of the microplate were merged, filtered and washed as described above, frozen at −20 °C and then lyophilized for 2 days in an Alpha 1-2 LDPlus freeze-dryer (Martin Christ, Germany) at −55 °C (condenser temperature) and 0.01 mbar pressure. The dried biomass was also used to calculate the cell dry weight (CDW). In order to obtain reliable dry cell weight data, the biomass grown on the walls of the wells was also collected and measured in contrast to that, used for FTIR and GC analysis.

### Lipid extraction

Direct transesterification was performed according to Lewis et al. [26] with modifications for lipid extraction from fungal biomass: 2 mL screw-cap polypropylene (PP) tubes were filled, in three technical replicates, with 30 ± 3 mg freeze dried fungal biomass, 250 ± 30 mg (710–1180 μm diameter) acid-washed glass beads (Sigma-Aldrich, USA) and 600 µL methanol. The fungal biomass was disrupted in a FastPrep-24 high-speed benchtop homogenizer (MP Biomedicals, USA) at 6.5 m s^−1^, for 1 min cycle length and 6 cycles. The disrupted fungal biomass was transferred into glass reaction tubes by washing the PP tube with 2400 µL methanol–chloroform–hydrochloric acid solvent mixture (7.6:1:1 v/v). Twenty microliters from a 25 mg mL^−1^ tridecanoic acid (C13:0, Sigma-Aldrich, USA) internal standard solution in methanol was added to the glass reaction tubes. The reaction mixture was vortexed for 10 s and incubated at 90 °C for 1 h, followed by cooling to room temperature and addition of 1 mL distilled water. The fatty acid methyl esters (FAMEs) were extracted by the addition of 2 mL hexane–chloroform (4:1 v/v) followed by 10 s vortex mixing. The reaction tubes were centrifuged at 3000*g* for 10 min at 4 °C and the upper hexane phase was collected in glass tubes. The hexane–chloroform extraction was performed thrice. Subsequently, the solvent was evaporated under nitrogen at 60 °C and FAMEs were dissolved in 1.5 mL hexane containing 0.01% butylated hydroxytoluene (BHT, Sigma-Aldrich, USA). The extracted non-lipid cell compounds (insoluble in hexane) were removed after centrifugation in Eppendorf tubes at 15,000*g* for 5 min at 4 °C. The FAMEs dissolved in hexane were transferred to GC vials containing small amount of anhydrous sodium sulfate.

### GC fatty acid analysis

Analysis of the extracted FAMEs was performed in a HP 6890 gas chromatograph (Hewlett Packard, USA) equipped with a SGE BPX70, 60.0 m × 250 µm × 0.25 µm column (SGE Analytical Science, Australia) and flame ionization detector (FID). Helium was used as a carrier gas. The runtime was 36.3 min with an initial oven temperature of 100 °C, which was increased steadily to 220 °C (4.3 min to 170 °C, then 20 min to 200 °C and 12 min to 220 °C). The injector temperature was 280 °C and 1 µL was injected in split mode (50:1 split ratio). FAMEs were identified with a C4–C24 FAME standard mixture (18919-1AMP, Supelco, USA) dissolved in hexane, and were quantified by the C13:0 internal standard and relative response factors (RRF) calculated from 5-point calibration curves of the individual FAMEs in the standard mixture.

### Data analysis

The FTIR spectra of fungal biomass were preprocessed in the following way: (1) technical replicates (630 spectra in total) were averaged resulting in 210 average spectra, (2) second derivative spectra were obtained by the Savitzky–Golay algorithm [27] using windows size 9 and a second degree polynomial, (3) Extended Multiplicative Signal Correction (EMSC), an MSC model extended by a linear and quadratic component, was employed on the combined spectral range comprising of 3100–2800 and of 1800–500 cm^−1^ spectral regions [28]. These spectral regions were selected since they contain bands distinctive for fungi [18, 29].

Principal component analysis (PCA) was applied in order to evaluate the differentiation ability of FTIR analysis between fungal species, cultivation temperature and time, and to compare sample variation pattern in FTIR and GC data concerning fatty acid composition. The preprocessed FTIR spectra (2nd derivative and EMSC) were used in the 3100–2800 cm^−1^ region, while the GC fatty acid data were autoscaled.

Partial least square regression (PLSR) was used to establish calibration models for fatty acid parameters of fungal biomass. In order to establish such models a data set of GC reference measurements (responses) were used as a Y matrix, which was regressed onto an X matrix containing FTIR measurements of fungal biomass (predictors). Fatty acid parameters included total lipid content of biomass and fatty acid compositional data such as saturated fatty acids (SAT), monounsaturated fatty acids (MUFA), polyunsaturated fatty acids (PUFA), unsaturation index and relevant single fatty acids. From a total of 210 averaged FTIR spectra, nine samples belonging to early growth phase of the fungi were excluded (3 biological replicates each of *M. circinelloides* 20 °C on day 1, *P. glabrum* 20 °C on day 2, and *P. glabrum* 30 °C on day 1). The remaining 201 average FTIR spectra were used for the PLSR. Reference GC fatty acid dataset was built on the measurement of 67 fungal samples, each of them measured in three technical replicates and then results were averaged. Therefore, one averaged GC fatty acid composition was used in the regression for three corresponding biological replicate FTIR spectra. In order to optimize the number of PLSR principal components (PCs), cross-validation (CV) was performed, where cross validation segments were defined by days. All spectra from samples obtained on the same day were removed in turn and used for validating the model established on the rest of the data. A root-mean-square error (RMSE) was calculated for models with 1–25 components. The optimal model was the one with the lowest number of PCs having insignificantly higher RMSE than the model with the minimum RMSE.

The Unscrambler, V10.3 (CAMO, Norway) and Matlab, V8.5 (The Mathworks, USA) software were used to perform the data analysis.

## Results and discussion

### Growth and lipid accumulation of oleaginous fungi in Duetz-MTPS

**Fig. 1:**
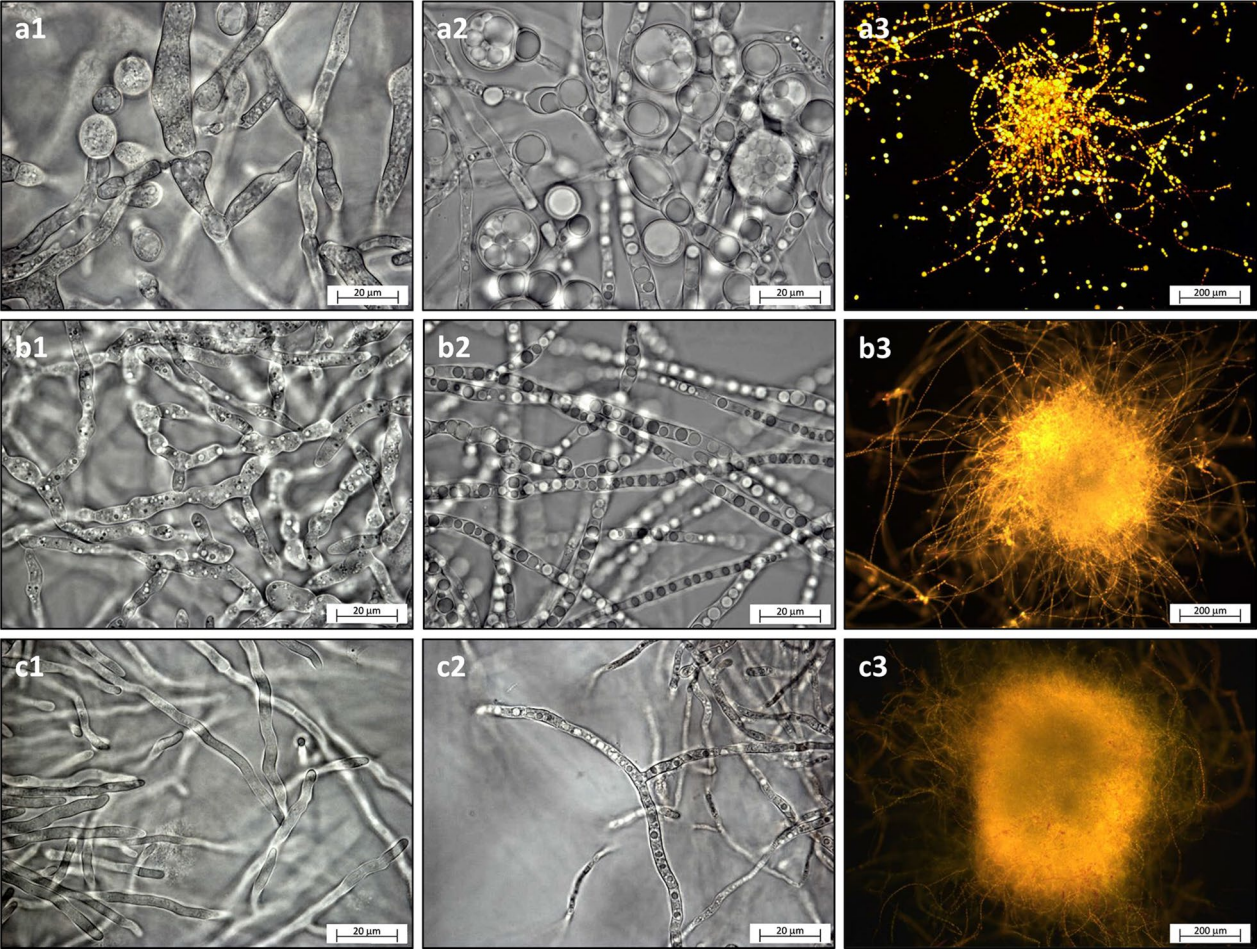
Bright-field (**a1**–**c2**) and fluorescent microscopy (**a3**–**c3**) images of *M. circinelloides* (**a**), *U. isabellina* (**b**) and *P. glabrum* (**c**). Images (**a1**–**c1**) show fungal hyphae before or at the beginning of lipogenesis (day 1–2) while images (**a2**–**c3**) were captured from hyphae filled with distinct lipid bodies (day 3–6)

The growth of dimorphic fungus *M. circinelloides* resulted in clumped mycelium mixed with dispersed yeast-like cells, while *U. isabellina* and *P. glabrum* grew in pellets of approx. 0.5–2 mm in diameter (Fig. 1). Maximum lipid bodies’ diameter were 17, 5 and 2.5 µm respectively (Fig. 1). It is worth mentioning that filling volume in the 24-well MTP should not exceed 2.5 mL at 300 rpm agitation speed in order to avoid the contact of fermentation broth with sandwich cover, which potentially leads to cross-contamination.

**Fig. 2:**
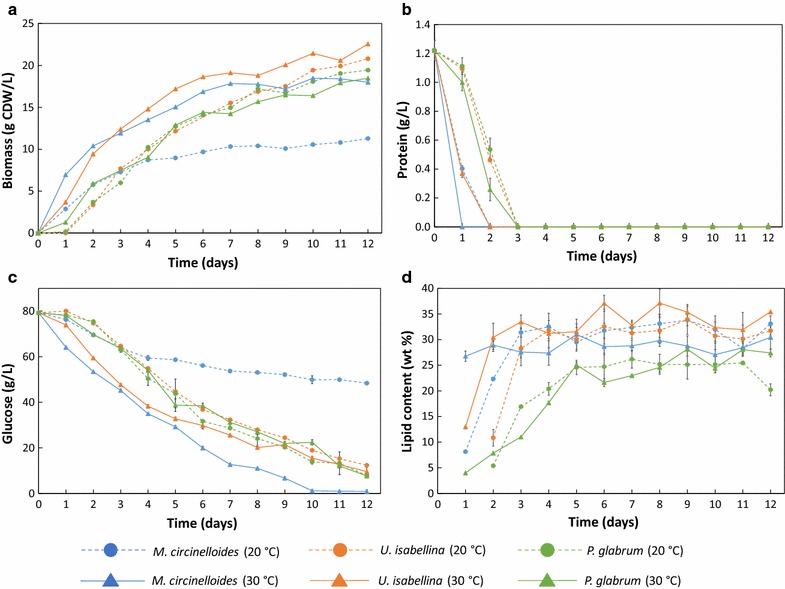
Biomass (**a**), protein (**b**) and glucose (**c**) concentrations in cultivation medium and lipid content (**d**) of *M. circinelloides*, *U. isabellina* and *P. glabrum* cultivated at 20 and 30 °C in the Duetz-MTPS (n = 3, *error bars* = SD)

For all three species, initial growth rate was higher at 30 °C than at 20 °C (Fig. 2a). Final biomass concentration (18–23 g L^−1^) was similar for the three fungi at both temperatures. The exception was *M. circinelloides* grown at 20 °C, which consumed less than half of the initial glucose and reached only 11 g L^−1^ biomass. However, at 30 °C, which is the optimum growth temperature for this species [30], *M. circinelloides* showed glucose depletion after 10 days of cultivation (Fig. 2c). Proteins from yeast extract were depleted within 1–3 days of cultivation for the three studied fungi (Fig. 2b), however growth continued at a reduced rate after nitrogen depletion. This result is in agreement with the study of Tang et al. [31], where the sole N-source, i.e., ammonium tartrate was depleted by *M. circinelloides* within 9–10 h, while growth continued until 60–80 h. *M. circinelloides* and *U. isabellina* reached 31–37% lipid content of the biomass, while *P. glabrum* accumulated 26–28% lipid (Fig. 2d). Further fermentation results can be found in Additional file 1: Table S1 and Figure S1.

**Fig. 3:**
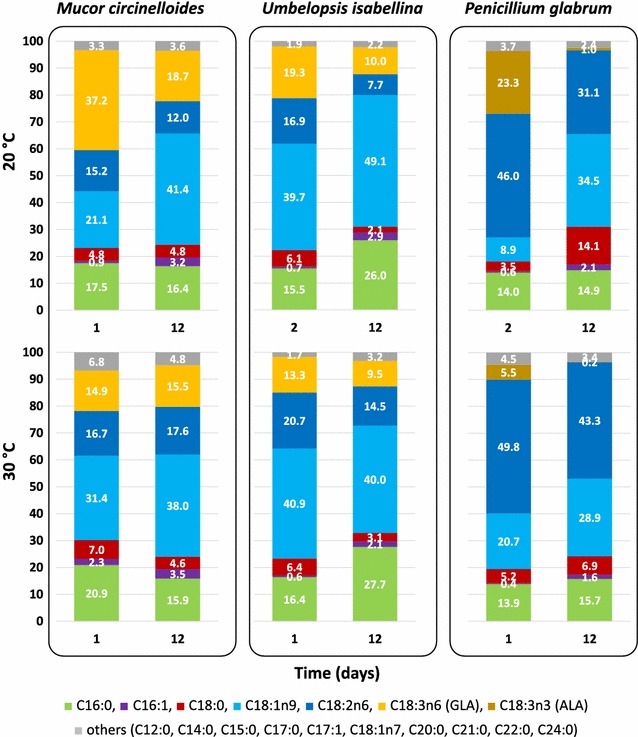
Fatty acid composition (%) of *M. circinelloides*, *U. isabellina* and *P. glabrum* cultivated at 20 °C and at 30 °C at first and last day of sampling in the Duetz-MTPS. Values represent mean value of three extraction—GC replicate measurements. Coefficient of variation was less than 2% for main fatty acids

*M.* *circinelloides* and *U. isabellina* produced unsaturated fatty acids up to 18 carbon chain length gamma-linolenic acid (C18:3n6 or GLA), while *P. glabrum* produced unsaturated fatty acids up to alpha-linolenic acid (C18:3n3 or ALA) (Fig. 3). These results are in agreement with previous studies, showing that more advanced fungi (Ascomycota and Basidiomycota) produce the n-3 isomer of the C18 trienoic fatty acid, while basal fungi (Mucoromycotina, Chytridiomycota) produce the n-6 isomer [32, 33]. After 12 days cultivation, higher content of oleic acid (C18:1n9), GLA (*M. circinelloides* and *U. isabellina*) and ALA (*P. glabrum*) was observed at 20 °C than at 30 °C, while linoleic acid (C18:2n6, LA) content was higher at 30 °C than at 20 °C for all the three studied fungi. The PUFA content in the oil, including LA, GLA and ALA rapidly decreased after the transition from exponential growth to stationary phase and remained relatively stable afterwards. In the stationary phase of *M. circinelloides* and *U. isabellina,* oleic acid content tended to increase concomitant with decrease in saturated fatty acids (C16:0, palmitic acid and C18:0, stearic acid) (Additional file 1: Figure S2).

### FTIR spectroscopy of oleaginous filamentous fungi

**Fig. 4:**
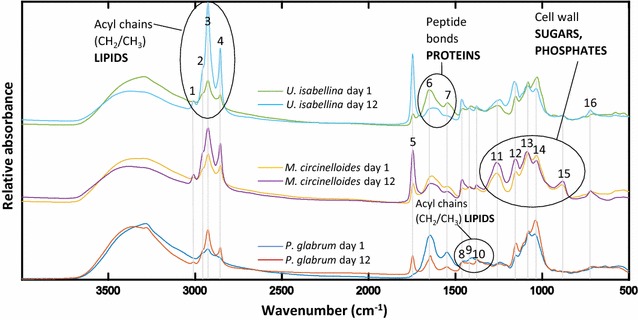
FTIR spectra of *P. glabrum*, *M. circinelloides*, *U. isabellina* (30 °C) after 1 and 12 days of cultivation

The FTIR spectra of *M. circinelloides*, *U. isabellina* and *P. glabrum* biomass after 1 and 12 days of cultivation are shown in Fig. 4. Assigned spectral bands of the FTIR spectrum and their respective functional groups are listed in Additional file 1: Table S2. In the infrared spectrum, cellular lipids were represented by several peaks, related to different lipid functional groups: (I) peaks in the regions 3050–2800 cm^−1^ (peaks Nº 1–4 in Fig. 4), 1500–1300 cm^−1^ (Nº 8–10) and at 725 cm^−1^ (Nº 16) are related to lipid acyl chains, (II) peak around 3008 cm^−1^ (Nº 1) corresponds to =C–H stretching and gives indication about the lipid unsaturation index [14, 15, 22], (III) the peak at 1745 cm^−1^ (Nº 5) is related to the ester carbonyl bond, which represents the vast majority of the total lipid content in the cell [14, 22]. We observed a relative increase with the cultivation time for all lipid related bands in mid-IR spectra of all studied fungi, indicating that during cultivation in nitrogen limited medium, the main biochemical changes in fungi were related to intracellular lipid accumulation. As a consequence, the relative absorbance of protein-related peaks between 1690 and 1500 cm^−1^, which are mainly influenced by the amide I (peak Nº 6 in Fig. 4) and amide II bands (Nº 7) [8], decreased during lipid accumulation.

Temporal-dependent changes in complex polysaccharide region (1240–900 cm^−1^) related to phosphate groups (nucleic acids, polyphosphates, phospholipids), as well as C–O and C–O–C absorption peaks of the cell wall polysaccharides (chitin, chitosan, β-glucan, mannan, etc.), were also observed for all fungi. The appearance of the strong peaks around 1260 (peak Nº 11 in Fig. 4) and 880 cm^−1^ (Nº 15) in *M. circinelloides* spectra indicated an increase in polyphosphate content in its cell wall, as reported previously [34]. The *U. isabellina* IR spectra showed an absorbance increase at 1240–1250 cm^−1^, that may also indicate a polyphosphate content increase, but this increase was less prominent than for *M. circinelloides.* Relative to amide I peak, the β (1,3)-glucan peaks (at 1150 and 1080 cm^−1^) (peaks Nº 12 and 13 in Fig. 4) and the C–O stretching peak around 1033 cm^−1^ (Nº 14) increased as well with the cultivation time for all studied fungi. Such changes in the polysaccharide region of IR spectra show that the cell wall composition changes during lipid accumulation. We can hypothesize that an increase of β (1,3)-glucan peaks corresponds to an increase in the cell wall thickness which goes along with lipid accumulation. Indeed, the homogenization of lipid-rich fungal biomass used for FTIR required long time, while that used for GC analysis required the use of hydrochloric acid and harsh mechanical pretreatment (bead beating) [34]. Ami et al. [8] and Signori et al. [10] observed similar cell wall changes in FTIR spectra during oleaginous yeast lipogenesis.

**Fig. 5:**
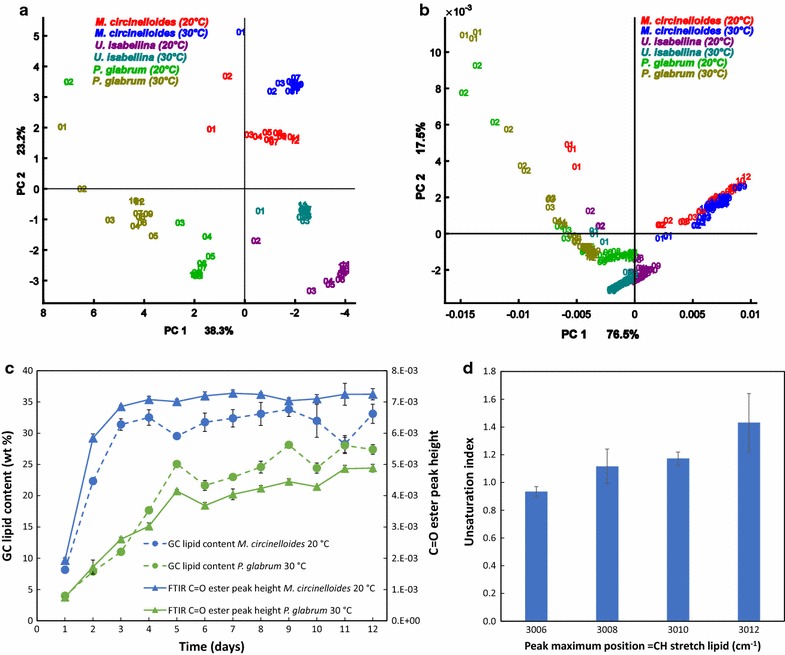
Exploratory analysis of FTIR data and comparison with GC reference data. **a** First and second scores (PC1, PC2) in PCA of the auto-scaled GC fatty acid data (PC1 axis is reversed in the GC score plot). **b** First and second scores in PCA of the preprocessed FTIR spectra in the spectral range of 3100–2800 cm^−1^. **c** Total lipid content measured by reference GC method and followed by the C=O ester peak height from the pre-processed FTIR spectra (n = 3, *error bars* = SD). **d** Relationship between unsaturation indices and position of the =C–H stretching bond peak maxima (cm^−1^) in FTIR spectra. Unsaturation index = [Σ(% monoene + 2× % diene + 3× % triene)]/100 [35]

PCA was performed on FTIR spectra and GC fatty acid data to evaluate temporal-, species- and cultivation temperature specific differences related to lipid production. The score plot of the first and second component of PCA of the FTIR data shows clustering according to the fungal species and cultivation temperatures (Fig. 5). The sample variation pattern in GC (Fig. 5a) and FTIR (Fig. 5b) score plots show similar tendencies and demonstrate that the main changes in fatty acid composition occur during the transition from growth phase (day 1–3) to the lipid accumulation phase (day 3–12). After 3 days of fermentation the fatty acid composition stabilizes. Furthermore, both GC and FTIR score plots indicate that reaching the stable fatty acid composition took longer for *P. glabrum* than for *M. circinelloides* and *U. isabellina* due to lower growth rate of this species. Biological replicate samples, which are marked by the same names and colors in the FTIR score plot, are close to each other. Variability between technical and biological replicates of the FTIR measurements were quantified by Pearson correlation coefficient (Additional file 1: Table S3). Technical and biological sample variability were of the same order, and two orders lower than variation between samples during the 12 days of cultivation. This demonstrates that the Duetz-system is a suitable high-throughput platform for the reproducible cultivation of filamentous fungi and that HTS-FTIR spectroscopy is a suitable high-throughput platform for the reproducible monitoring of lipogenesis of oleaginous fungi.

The total lipid content of the biomass was monitored using the C=O ester peak height (1745 cm^−1^) of the preprocessed FTIR spectra. Total lipid trends for fungal biomass were assessed by the C=O ester peak height and compared to reference GC total lipid measurement (Additional file 1: Figure S3). For example, Fig. 5c shows that the GC and FTIR based lipid content of biomass curves for *M. circinelloides* (20 °C) and *P. glabrum* (30 °C) are well correlated. The fluctuation observed in the GC total lipid data may be due to the extraction–transesterification protocol for GC measurement, which is more prone to measurement errors than the FTIR measurement, which is done on almost intact cells. Although FTIR total lipid data cannot be used for absolute quantification without calibration to reference analysis, it provides a reliable and rapid qualitative method to monitor lipid accumulation during the cultivation of oleaginous species.

The position of the olefinic group around 3010 cm^−1^ is known to be related to the degree of unsaturation of fatty acids. It has been shown that a shift toward higher wavenumber suggests higher degree of unsaturation [14, 15]. In this study the peak position at 3006 cm^−1^ corresponded to an unsaturation index of 0.93 ± 0.04, while peak position 3012 cm^−1^ was described by an unsaturation index of 1.43 ± 0.21. Peak maxima between 3008 and 3010 cm^−1^ corresponded to an unsaturation index of 0.99–1.22 (Fig. 5d).

For the quantitative prediction of fatty acid composition and total lipid content of biomass PLSR models were established by using FTIR spectra (Additional file 2: Table S5) as X variables (or predictors) and GC fatty acid data (Additional file 3: Table S6) as Y variables (or responses). For seven of the fatty acid parameters, excellent calibration results were achieved with regression coefficients R^2^ above 0.91 and for three fatty acid parameters, good models were obtained with R^2^ between 0.86 and 0.89 (Table 1). Residual predictive deviation of cross-validation (RPD_CV_) values were good to acceptable between 2.6 and 5.1. The slightly lower prediction ability for total lipid content might be due to the high variability in GC quantification (Fig. 2d). The models for MUFA and oleic acid (C18:1n9) had a high complexity involving a higher number of PLS factors compared to the other models. This high complexity may involve some degree of over-fitting. The predictions of linolenic acid and unsaturation index are shown in Additional file 1: Figure S4, where the predicted values are plotted against the measured values for both prediction and validation results. Since both prediction and validation results show the same high prediction ability, we can conclude that the calibration models are stable. Prediction result including all 210 samples can be found in Additional file 1: Table S4.

**Table 1:** PLS regression results between HTS-FTIR and GC fatty acid measurements (N = 201)

Fatty acid	Range	Mean	Standard deviation	R^2a^	RMSECV^b^	RPD_CV_^c^	PLS factors
C16:0	13.4–31.9	20.2	6.1	0.94	1.5	4.0	6
C18:0	2.1–14.4	6.3	3.2	0.94	0.8	4.2	6
C18:1n9	25.4–49.1	37.4	5.4	0.89	1.8	3.0	21
C18:2n6	7.6–48.1	20.8	11.5	0.96	2.3	5.0	7
C18:3n6	0.0–22.3	9.1	7.2	0.96	1.4	5.1	3
SAT	22.4–39.2	29.0	4.5	0.87	1.6	2.8	6
MUFA	27.1–52.6	40.7	5.6	0.93	1.5	3.9	21
PUFA	15.9–50.5	30.3	8.1	0.93	2.2	3.7	7
Unsaturation index	0.89–1.33	1.11	0.13	0.95	0.03	4.5	9
Total lipid	7.9–37.1	27.8	6.1	0.86	2.3	2.6	4

^a^R^2^, cross-validated squared correlation coefficient

^b^RMSECV, root mean square error of cross validation

^c^RPD_CV_, residual predictive deviation of cross-validation (standard deviation/RMSECV)

## Conclusions

In this study, we have examined a high-throughput approach for the cultivation and monitoring of oleaginous fungi. For this purpose, a Duetz microtiter plate system was combined with rapid FTIR spectroscopy of fungal biomass. First, the microcultivation performance was evaluated for the studied fungi. Biological replicate cultivations in the 24 well deep well microtiter plate showed excellent reproducibility based on glucose consumption (pooled standard deviation = 1.1 g L^−1^ glucose) and FTIR spectra of biomass (average Pearson correlation coefficient = 0.9994). Fungal cultures with high biomass concentrations (up to 23 g L^−1^ CDW) and high lipid content (up to 35%) were achieved in the Duetz-MTPS.

Evaluation of the FTIR spectra of fungi during fermentation resulted in lipid accumulation curves that followed very similar trends to total lipid curves obtained by reference GC quantification. In general, the lipid accumulation curves on the basis of the FTIR C=O ester peak showed smaller day-to-day variations and thus are more plausible. For quantitative purposes, the FTIR spectral data of biomass were calibrated versus GC fatty acid data. Summed fatty acid parameters (SAT, MUFA, PUFA, total fat, unsaturation index) and main individual fatty acids were predicted with high precision (R^2^ between 0.86 and 0.96, RPD_CV_ between 2.6 and 5.1). Sample preparation for HTS-FTIR measurement can be fully automated by robotics to further increase precision and throughput. We therefore conclude that HTS-FTIR spectroscopy is a simple, rapid tool for screening or at-line monitoring the cultivation of oleaginous species.

## Additional files

**Additional file 1.** **Figures S1–S4**, **Tables S1–S4.** Additional figures and tables.

**Additional file 2: Table S5.** HTS-FTIR spectra of biomass.

**Additional file 3: Table S6.** GC fatty acid composition.

**Additional file 4: Table S7.** HPLC glucose concentration.

## Abbreviations

Duetz-MTPS: Duetz microtiter plate system
EMSC: extended multiplicative signal correction
HTS: high-throughput screening
FTIR: Fourier transform infrared spectroscopy
MUFA: monounsaturated fatty acids
SAT: saturated fatty acids
PUFA: polyunsaturated fatty acids
PCA: Principal Component Analysis
PLSR: partial least squares regression
