# Supplementary material for: Microtiter plate cultivation of oleaginous fungi and monitoring of lipogenesis by high-throughput FTIR spectroscopy
Source: Microb Cell Fact. 2017 Jun 9;16:101. doi: 10.1186/s12934-017-0716-7 (PMC5466753; doi:10.1186/s12934-017-0716-7)
Supplement: Supplementary file 1 — Additional file 1. Figures S1–S4, Tables S1–S4. Additional figures and tables. [file 12934_2017_716_MOESM1_ESM.docx]

**Supplementary Material**

**Microtiter plate cultivation of oleaginous fungi and monitoring of lipogenesis by high-throughput FTIR spectroscopy**

Gergely Kosa *,Achim Kohler, Valeria Tafintseva, Boris Zimmermann, Kristin Forfang, Nils Kristian Afseth, Dimitrios Tzimorotas, Kiira S. Vuoristo, Svein Jarle Horn, Jerome Mounier, Volha Shapaval

*Corresponding author:

**Gergely Kosa**

Faculty of Science and Technology

Norwegian University of Life Sciences

Postbox 5003, 1432 Ås, Norway

Email: gergely.kosa@nmbu.no

**Table of contents: Page**

Fermentation results S2

Fatty acid composition of fungi S3

Peak assignment in the FTIR spectra of fungi S4

Variability in FTIR spectra at different levels S5

Total lipid data by GC and FTIR spectroscopy S6

Predicted vs. measured for linoleic acid and unsaturation index S7

PLSR results of fatty acid properties (N=210) S7

**Table S1** Maximum measured value of biomass concentration (CDW, g L^-1^), lipid content of cell dry weight (wt %), total lipid concentration (g L^-1^), and GLA concentration (mg L^-1^) in the fermentation broth. Yield of biomass (g g^-1^) and yield of total lipids (g g^-1^) per glucose carbon source

| Strain | Temperature  (°C) | Biomass  (g L^-1^) | Lipid content  (wt %) | Total lipid  (g L^-1^) | GLA  (mg L^-1^) | Biomass/Glucose  (g g^-1^) | Total lipid/Glucose  (g g^-1^) |
| --- | --- | --- | --- | --- | --- | --- | --- |
| *M. circinelloides*  *VI 04473* | 20 | 11.3 | 34 | 3.7 | 698 | 0.29 | 0.12 |
|  | 30 | 18.5 | 31 | 5.5 | 851 | 0.17 | 0.05 |
| *M. isabellina*  *UBOCC-A-101350* | 20 | 20.8 | 34 | 6.6 | 662 | 0.29 | 0.09 |
|  | 30 | 22.6 | 37 | 8.0 | 760 | 0.28 | 0.11 |
| *P. glabrum*  *FRR 4190* | 20 | 19.4 | 26 | 4.9 | - | 0.26 | 0.07 |
|  | 30 | 18.5 | 28 | 5.1 | - | 0.23 | 0.08 |


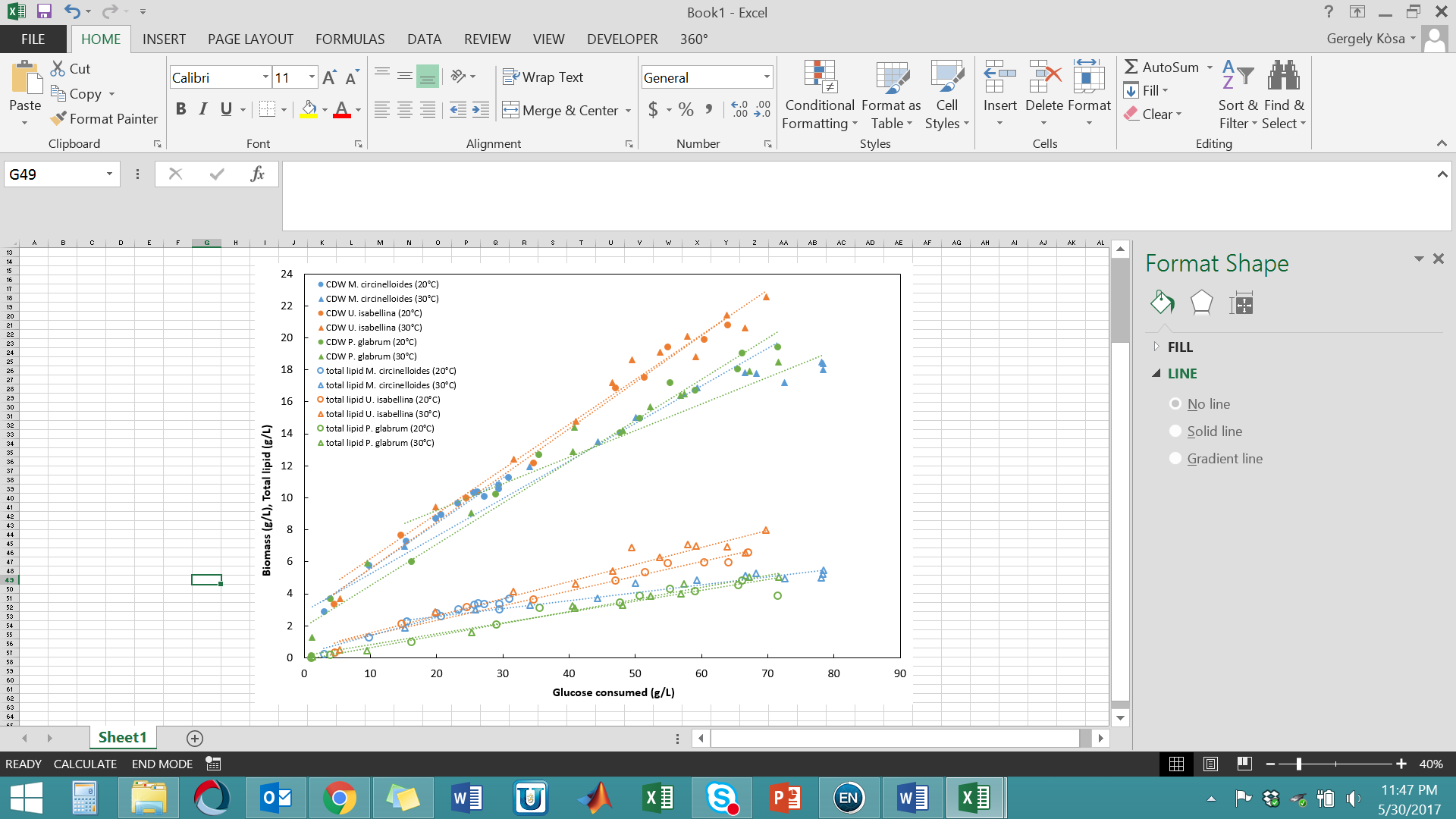


**Figure S1** Biomass concentration (g L^-1^) and total lipid content of biomass (g L^-1^) as a function of consumed glucose (g L^-1^). Yield of biomass (g g^-1^) and yield of lipids (g g^-1^) were calculated as the slope of linear regression lines of biomass and total lipid versus the amount of consumed glucose

**Figure S2** Fatty acid composition (%) of *M. circinelloides*, *U. isabellina* and *P. glabrum* cultivated at 20 °C and at 30 °C during cultivation for 12 days in the Duetz-MTPS. Values represent mean value of three extraction – GC replicate measurements. Coefficient of variation was less than 2 % for main fatty acids

**Table S2** Peaks assignment in the FTIR spectra of microbial biomass. Peaks *1-5*, *8-10* and *16* are characteristic to lipids

| Peak Nr. | Wavenumber (cm^-1^) | Peak assignment | Reference |
| --- | --- | --- | --- |
| *1* | *3008* | *=C-H stretching* | [1] |
| *2* | *2953* | *-C-H (CH_3_) stretching (asym)* | [1] |
| *3* | *2924* | *-C-H (CH_2_) stretching (asym)* | [1] |
| *4* | *2853* | *-C-H (CH_2_) stretching (sym)* | [1] |
| *5* | *1745* | *-C=O (ester) stretching* | [1] |
| 6 | 1695-1637 | -C=O stretching, Amide I | [2] |
| 7 | 1550-1520 | N-H bending and C-N stretching, Amide II | [2] |
| *8* | *1465* | *-C-H (CH_2_, CH_3_) bending (scissoring)* | [1] |
| *9* | *1415* | *C-H rocking* | [2] |
| *10* | *1377* | *-C-H (CH_3_) bending (sym)* | [1] |
| 11 | 1240-1260 | P=O stretching | [3] |
| 12 | 1150 | β (1,3)-glucans | [4] |
| 13 | 1080 | β (1,3)-glucans | [4] |
| 14 | 1033 | C-O stretching | [5] |
| 15 | 880 | P-O-P stretching | [6] |
| *16* | *720* | *CH_2_ rocking, bending* | [1] |

**References:**

1. Guillen MD, Cabo N: Relationships between the composition of edible oils and lard and the ratio of the absorbance of specific bands of their Fourier transform infrared spectra. Role of some bands of the fingerprint region. *Journal of Agricultural and Food Chemistry* 1998, **46:**1788-1793.

2. Kohler A, Afseth NK, Jørgensen K, Randby Å, Martens H: Quality Analysis of Milk by Vibrational Spectroscopy. In *Handbook of Vibrational Spectroscopy.* John Wiley & Sons, Ltd; 2006.

3. Davis R, Mauer L: Fourier transform infrared (FT-IR) spectroscopy: a rapid tool for detection and analysis of foodborne pathogenic bacteria. *Current research, technology and education topics in applied microbiology and microbial biotechnology* 2010, **2:**1582-1594.

4. Signori L, Ami D, Posteri R, Giuzzi A, Mereghetti P, Porro D, Branduardi P: Assessing an effective feeding strategy to optimize crude glycerol utilization as sustainable carbon source for lipid accumulation in oleaginous yeasts. *Microb Cell Fact* 2016, **15:**75.

5. Isleten-Hosoglu M, Gultepe I, Elibol M: Optimization of carbon and nitrogen sources for biomass and lipid production by Chlorella saccharophila under heterotrophic conditions and development of Nile red fluorescence based method for quantification of its neutral lipid content. *Biochemical Engineering Journal* 2012, **61:**11-19.

6. Carosio F, Alongi J, Malucelli G: Layer by Layer ammonium polyphosphate-based coatings for flame retardancy of polyester–cotton blends**.** *Carbohydrate Polymers* 2012, **88:**1460-1469.

**Table S3** Variability in FTIR spectra at different levels. (1-PCC) x 10^4^. PCC: Pearson Correlation Coefficient

| Data set | *M. circinelloides* 20 °C | *M. circinelloides* 30 °C | *U.*  *isabellina*  20 °C | *U.*  *isabellina*  30 °C | *P.*  *glabrum*  20 °C | *P.*  *glabrum*  30 °C |
| --- | --- | --- | --- | --- | --- | --- |
| Technical replicates  (3 spots in 384 well HTS plate) | 1.4 | 0.8 | 5.4 | 2.1 | 2.9 | 4.0 |
| Biological replicates (3 wells in the 24 well microplate) | 3.6 | 3.0 | 4.6 | 2.7 | 10.9 | 10.5 |
| run | 322.7 | 75.2 | 359.2 | 212.8 | 326.1 | 393.3 |

**Figure S3** Total lipid content measured by reference GC method and monitored by the ester peak height of FTIR spectra (n=3, error bars = SD)

**Figure S4** PLS regression results between reference GC-FID data and predicted FTIR data. **a** linoleic acid (C18:2n6c); **b** unsaturation index. Blue points represent the calibration fit obtained from the model on FTIR and GC data (closer to the target diagonal line), while red points show the predictions obtained in the more realistic cross-validation

**Table S4** PLS regression results with all samples included (N=210)

| Fatty acid | Range | Mean | Standard  deviation | R^2a^ | RMSECV^b^ | RPD_CV_^c^ | PLS factors |
| --- | --- | --- | --- | --- | --- | --- | --- |
| C16:0 | 13.4-31.9 | 19.9 | 6.0 | 0.84 | 2.4 | 2.5 | 3 |
| C18:0 | 2.1-14.4 | 6.2 | 3.2 | 0.91 | 1.0 | 3.2 | 9 |
| C18:1n9 | 8.9-49.1 | 36.5 | 6.8 | 0.87 | 2.5 | 2.7 | 17 |
| C18:2n6 | 7.6-49.8 | 21.5 | 12.2 | 0.94 | 3.1 | 4.0 | 6 |
| C18:3n6 | 0.0-37.2 | 9.2 | 8.0 | 0.95 | 1.7 | 4.7 | 3 |
| SAT | 20.0-39.2 | 28.7 | 4.6 | 0.83 | 1.9 | 2.4 | 6 |
| MUFA | 10.4-52.6 | 39.7 | 7.2 | 0.75 | 3.7 | 2.0 | 10 |
| PUFA | 15.9-69.5 | 31.5 | 10.0 | 0.93 | 2.7 | 3.7 | 11 |
| unsaturation index | 0.89-1.73 | 1.13 | 0.16 | 0.90 | 0.05 | 3.1 | 7 |
| total lipid | 4.0-37.1 | 26.8 | 7.5 | 0.86 | 2.8 | 2.6 | 4 |

^a^ R^2^, cross-validated squared correlation coefficient

^b^ RMSECV, Root Mean Square Error of Cross Validation

^c^ RPD_CV_, Residual predictive deviation of cross-validation (standard deviation/RMSECV)
